# N6-methyladenosine-dependent modification of circGARS acts as a new player that promotes SLE progression through the NF-κB/A20 axis

**DOI:** 10.1186/s13075-022-02732-x

**Published:** 2022-02-04

**Authors:** Xingwang Zhao, Rui Dong, Longlong Zhang, Junkai Guo, Ying Shi, Lan Ge, Juan Wang, Zhiqiang Song, Bing Ni, Yi You

**Affiliations:** 1 Department of Dermatology, Southwest Hospital, Army Medical University (Third Military Medical University), Chongqing, China; 2 Chongqing International Institute for Immunology, Chongqing, China; 3 State Key Laboratory for Conservation and Utilization of Bio-Resources & Key Laboratory for Microbial Resources of the Ministry of Education, School of Life Science, Yunnan University, Kunming, China; 4 Department of Traditional Chinese Medicine, Southwest Hospital, Army Medical University (Third Military Medical University), Chongqing, China; 5 Department of Pathophysiology, College of High Altitude Military Medicine, Army Medical University (Third Military Medical University), Chongqing, China

**Keywords:** circGARS, miR-19a, A20, m6A, YTHDF2, Systemic lupus erythematosus

## Abstract

**Background:**

Certain circRNAs could be used as biomarkers to determine the risk of development and/or severity of systemic lupus erythematosus, and their new function in the regulation of gene expression has motivated us to investigate their role in SLE

**Methods:**

Experimental methods including qRT-PCR, RNA immunoprecipitation (RIP), pulldown, dual luciferase reporter assay, RNA interference and cell transfection, RNA fluorescence in situ hybridization, western blotting, and mass spectrometry were used to assessed circGARS (hsa_circRNA_0009000) for immune functions and defined mechanisms by which circGARS promotes the progression in SLE.

**Results:**

Our results demonstrated that the levels of circGARS was remarkably upregulated in SLE and correlated with clinicopathological features. CircGARS directly combined with microRNA-19a (miR-19a). Functionally, circGARS downregulated the expression of TNFAIP3 (A20, tumor necrosis factor alpha-induced protein 3) to mediate the activation of immune responses that were regulated by the nuclear factor-κB (NF-κB) pathway as a negative feedback mechanism. In addition, miR-19a regulated A20 (TNFAIP3) degradation by downregulating the expression of YTH N6-methyladenosine RNA-binding protein 2 (YTHDF2).

**Conclusions:**

The circGARS sponges miR-19a to regulate YTHDF2 expression to promote SLE progression through the A20/NF-κB axis and may act as an independent biomarker to help the treatment of SLE patients.

**Supplementary Information:**

The online version contains supplementary material available at 10.1186/s13075-022-02732-x.

## Background

Systemic lupus erythematosus (SLE) is a chronic and systemic autoimmune disease with diverse clinical manifestations and complicated disease progression. The pathogenesis of SLE are still largely unknown. A highly complicated interaction among various environmental factors and genetic susceptibility is most likely involved [1, 2]. Although mortality for patients with SLE has greatly declined, accurate diagnosis and better management of complications, especially the development of safer and more effective therapies, are still urgent. Therefore, there is an urgent need to gain an in-depth understanding the pathogenesis of SLE.

MicroRNAs (miRNAs) and long noncoding RNAs (lncRNAs) are two main kinds of noncoding RNAs that regulate the initiation and development of SLE [3, 4]. CircRNA (circRNA) is a novel non-coding RNA with a unique covalent closed-loop structure, which makes it an excellent diagnostic marker for SLE [5, 6]. In recent years, more functional circRNAs have been discovered using high-throughput sequencing analysis and bioinformatic methods, and circRNAs have been shown to play an important role in the progression of autoimmune diseases. Moreover, circRNAs have been revealed to act as microRNA (miRNA) sponges, participating in binding to RNA-binding proteins and protein translation [7–9]. Studies have reported that numerous circRNAs are associated with SLE, such as hsa_circ_0000479, hsa_circ_0068367, and hsa_circ_0044235 [10, 11]. However, the exact molecular mechanism remains to be elucidated.

m6A RNA modification is a widespread reversible dynamic modification in eukaryotic cells and is one of the most abundant mRNA nonterminal modifications. YTHDF2 protein can directly recognize m6A; the levels of YTHDF2 mRNA are decreased in peripheral blood from patients with SLE, which might be risk factors for SLE [12, 13]. TNF-α-induced protein 3 (TNFAIP3, also known as A20) is significantly downregulated in peripheral blood mononuclear cells (PBMCs) of SLE patients. It is one of the major SLE susceptibility genes involved in the negative regulation of inflammatory responses through modulation of the NF-κB pathway [14]. Genetic polymorphisms analysis suggests that the miR-17-92 cluster closely related with SLE of Chinese population [15]. The miR-17-92 cluster member miR-19a play an important role in multiple autoimmune patients. miR-19a inhibited expression of several targets including SOCS1 and A20 [16–18]. The regulatory effects of miR-19a on TNFAIP3 (A20) and NF-κB signaling have been reported, which involved in immune inflammatory responses of SLE [19–21]. Considering the role of m6A modification in regulating gene expression and immune response, the association between m6A modification and SLE remains to be clarified. It is possible that m6A modification may be involved in SLE etiology and participate in the initiation and progression of SLE [22]. However, there are relatively few studies on circRNAs in this area.

Herein, our aim was to analyze the molecular mechanisms of circRNAs in SLE patients. circGARS was specifically hyperexpression and closely related to the disease activities of SLE patients. To investigate whether the abnormal activation of the A20/NF-κB signaling pathway was regulated by circGARS, we studied how circGARS enhanced transcriptional levels by directly binding to m6A modification proteins, which might participate in the pathogenesis of SLE. We concentrated on the effect of circGARS competitively binding to miR-19a and regulating the expression of YTHDF2, which may regulate A20 degradation and eventually promote disease progression. Our study may provide a potential biomarker for SLE patients and/or a promising therapeutic target against SLE.

## Materials and methods

### Clinical samples

We collected 62 samples of SLE patients and healthy volunteers from the First Affiliated Hospital of Army Medical University from September 2017 to January 2020. Patients who met four or more American College of Rheumatology (ACR) SLE criteria, as revised in 1997, were included. Demographic, clinical and laboratory characteristics were recorded for each subject and disease activity was assessed using the Systemic Lupus Erythematosus Disease Activity Index (SLEDAI) at blood drawing. The information about the SLE patients can be found in Additional File 1: Table S1 and Additional File 2: Table S2.

### RNA extraction and RT-qPCR

The whole blood (10 ml) was collected in the anticoagulant tube from each subject, and PBMCs (peripheral blood mononuclear cells) were isolated by density-gradient centrifugation using Ficoll-Paque Plus (GE Healthcare Biosciences) within 4 h of collecting the samples. Total RNA was harvested and separated from PBMCs of samples via TRIzol reagent (Invitrogen). Extracted total RNA was reverse-transcribed to complementary DNA (cDNA) using Prime Script RT Master Mix (Takara, Japan) with random or oligo (dT) primers. SYBR Green SuperMix (Roche, Basel, Switzerland) was used for qRT-PCR. The relative expression levels were detected by the 2^−ΔΔCt^ method. The primers are listed in Additional File 3: Table S3.

### Ribonuclease R treatment

Two micrograms of total RNA from PBMCs was mixed with or without 3 U/μg ribonuclease R (Epicentre Technologies, Madison, WI, USA) at 37 °C for 20 min. Then, the samples were purified with an RNeasy MinElute Cleaning Kit (74204, Qiagen, Germany) and analyzed by RT-PCR. The stability of circGARS and linear GARS was determined.

### Fluorescence in situ hybridization (FISH)

RNA in situ hybridization was performed using specific probes for circGARS and miR-19a-5p. The circGARS probe for FISH was 5′-ATCCTTCTTATATGCCTTAC-3′. The experiment followed the manufacturer’s instructions for Alexa Fluor™ 488 Tyramide SuperBoost™ Kits by Riobio (Guangzhou, Guangdong, China). Confocal microscopy was used to better visualize the presence of circGARS and miRNA-19a-5p.

### PBMCs isolation and culture

1 × 10^6^ PBMC cells were cultured in RPMI Media 1640 (Invitrogen-Gibco, USA) supplemented with 10% fetal bovine serum (Invitrogen-Gibco, USA), 100 U/mL penicillin, 100 U/mL streptomycin (Invitrogen-Gibco, USA), and 1.5 mg/L glutamine and incubated at 37 °C with 5% CO_2_ for 24 h before transfection.

### Plasmid construction and cell transfection

Cells were separately transfected with Lipofectamine 3000 (Invitrogen, Carlsbad, CA). The circGARS overexpression plasmids and empty vector were constructed by GeneChem (Shanghai, China). The miR-19a-3p/5p mimics/inhibitor, miRNA-NC mimics/inhibitor, siRNA for circGARS, and siRNA-NC were chemically synthesized by Riobio (Guangzhou, Guangdong, China). The sequence of siRNA for circGARS was 5-ATGAGAAAGGGGTTGGATTGA-3. The transfection process lasted 48 h.

### Luciferase reporter gene assay

For the circGARS and miR-19a-5p luciferase reporter gene assays, the circGARS sequences containing wild-type miR-19a-5p predicted binding sites were inserted into the region directly downstream of a cytomegalovirus (CMV) promoter-driven firefly luciferase cassette in a pCDNA3.1 vector by GeneChem (Shanghai, China). For the YTHDF2 3′-UTR and miR-19a-3p luciferase reporter gene assay, the YTHDF2 3′-UTR sequences containing wild-type miR-19a-3p predicted binding sites were inserted into the region directly downstream of a T7 promoter-driven firefly luciferase cassette in a psiCHECK^TM^-2 vector (Promega, Madison, USA). All constructs were detected by sequencing. 2 × 10^5^ 293T cells were seeded into 24-well plates and co-transfected with a mixture of 1 μg of luciferase reporter and miRNA mimics and inhibitor. After 48 h of incubation, the firefly and Renilla luciferase activities were quantified using the Dual Luciferase Assay System (Promega, Madison, WI, USA).

### Quantification of the m6A modification

Concentrations of m6A in PBMC supernatants were analyzed by an Epiquik m6A RNA Methylation Quantification Kit (colorimetric) following the manufacturer’s instructions. The absorbance was measured at 450 nm using a microplate analyzer and the horizontal colorimetric value of m6A was measured according to the standard curve.

### Prediction of ceRNAs for circGARS

To explore the functions of the candidate disease stage-related circGARS, the potential miRNA binding sites were predicted. We used circMir1.0 software to identify circGARS-targeting miRNAs, which indicates a higher probability of being a putative ceRNA for circGARS.

### RNA-binding protein immunoprecipitation (RIP)

A Magna RIP RNA-Binding Protein Immunoprecipitation Kit (Millipore, Billerica, MA) was used to perform the RIP experiments according to the manufacturer’s instructions. Human AGO2 antibody (ab57113, Abcam, Cambridge, MA) was used for RIP. In brief, the PBMCs was harvested and dissolved on ice in the RIP lysis buffer for 30 min. After centrifugal collection, the supernatant was incubated with 30 μl Protein-G agarose beads (Roche, USA) and 8ul AGO2 antibodies. After overnight incubation, the complexes were centrifuged and then washed six times using washing buffer. The bead-bound proteins were analyzed by western blotting. Co-precipitated RNA was detected by qRT-PCR.

### RNA pulldown

The biotin-coupled RNA complex was pulled down by incubating the cell lysates with Pierce™ Streptavidin Magnetic Beads (Thermo Fisher Scientific, USA) as per the manufacturer’s protocol. The enrichment of circGARS in the capture fractions was detected by qRT-PCR analysis. The circGARS junction probe was 5′-CAGCACATCCAACAATCTCA-3′, and the control probe was 5′-TTGTACTACACAAAAGTACTG-3′ (ordered from Sangon Biotech, Shanghai, China). miRNAs were detected by RT-PCR. The probe sequences used are listed in Additional File 4: Table S4.

### Western blot analysis

RIPA lysis buffer (Beyotime, Shanghai, China) supplemented with protease inhibitors (P8340, Sigma-Aldrich) was used to lyse the total protein from PBMCs. The protein concentration was determined using a Braford Protein Assay Kit (Beyotime, Shanghai, China). Total cell lysates were separated by 10% sodium dodecyl sulfate polyacrylamide gel electrophoresis (SDS-PAGE) and then transferred to polyvinylidene fluoride membranes (Millipore, Bradford, MA, USA). Membranes were blocked with milk for 2 h on a shaker at room temperature followed by incubation with primary antibodies overnight at 4 °C. The primary antibodies used were mouse anti-Argonaute-2 (ab57113, Abcam, 1:1000), rabbit anti-A20 (5630S, Cell Signaling Technology, CST, 1:1000), rabbit anti-NF-κB (8242 T, CST, 1:1000), rabbit anti-p-NF-κB (3033 T, CST, 1:1000), rabbit anti-IkBα (9242S, CST, 1:1000), rabbit anti-p-IkBα (2859S, CST, 1:1000), rabbit anti-YTHDF2 (80014S, CST, 1:1000), rabbit anti-β-actin (4970S, CST, 1:1000), and GAPDH antibody (5174S, CST, 1:1000), which were used as controls. After washing with PBST three times, the blots were then incubated with horseradish peroxidase-coupled anti-rabbit antibody (7074S, CST, 1:1000) or anti-mouse antibody (7076S, CST, 1:1000) for 1 h at room temperature. The blot signal was examined by Pierce ECL western blotting Substrate (Thermo Fisher Scientific, USA) with a ChemiDoc™ Touch Imaging System (Bio-Rad, USA) according to the manufacturer’s recommendations. The integrated density values were calculated using Quantity One software (Bio-Rad, USA).

### Statistical analysis

GraphPad Prism version 8.0 (GraphPad Software, La Jolla, CA) was used to prepare graphs and presented as the mean ± standard deviation. The difference was determined via the paired Student’s *t* test. All experiments mentioned above were repeated three times independently. A two-tailed *P* value < 0.05 was considered statistically significant.

## Results

### Identification and characterization of circGARS, a circRNA specifically and highly expressed in SLE

**Fig. 1:**
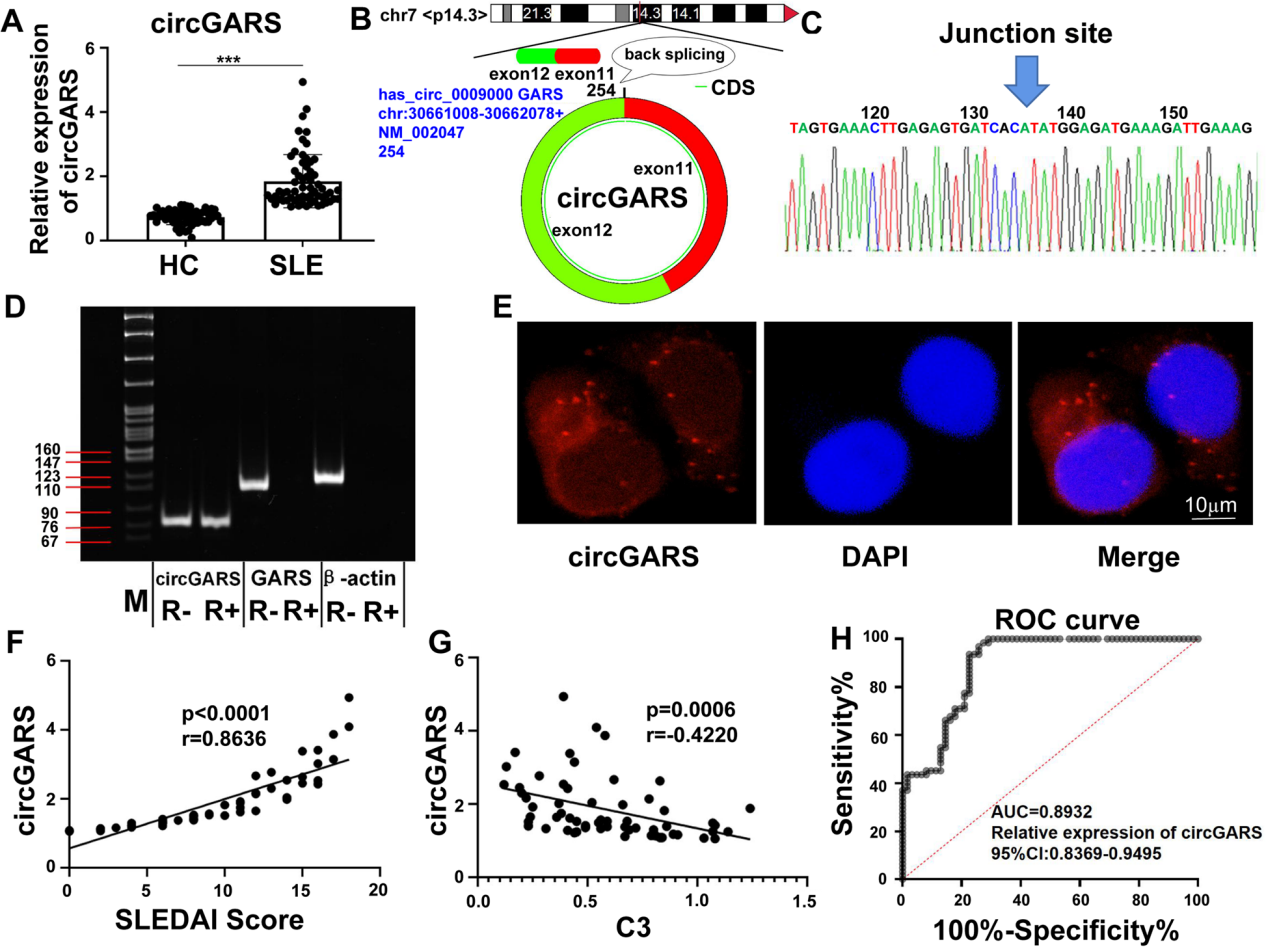
CircGARS is overexpressed in SLE and significantly related to disease activities. **a** Identification of circGARS with significant differences (*P* < 0.001) in SLE patients compared with healthy controls. **b** Structures of the circGARS genome and transcript. circGARS is produced by exons 11-12, and its genomic size was determined as included in the circBase database. **c** Verification of the junction point of circGARS by qRT-PCR followed by Sanger sequencing. **d** RNase R treatment determined the circular form of circGARS. And circGARS, rather than linear GARS or actin, could resist digestion by RNase R through PCR and an agarose gel electrophoresis assay. **e** FISH (fluorescence in situ hybridization) for circGARS. Nuclei were stained with DAPI (4′,6-diamidino-2-phenylindole). Scale bar: 10 μm. **f**, **g** Correlations between the expression of circGARS and the systemic lupus erythematosus disease activity index (SLEDAI) and complement C3 levels were analyzed. **h** ROC curve of relative circGARS expression for differentiating 62 patients with SLE from 62 healthy controls. The results are represented as the mean ± SD (*n* = 3). NS, no significance, ***P < 0.001

To identify circRNAs specifically functioning in SLE, we analyzed the circRNA RNA-seq data to identify circRNAs with significant differences (*P* < 0.01) between healthy controls and SLE patients [23]. We selected and identified 10 upregulated circRNAs and 10 downregulated circRNAs (|fold change| > 2, *P* < 0.01, Additional File 5: Fig. S1 and Additional File 6: Table S5) significantly related to SLE. The results showed that circGARS (known as hsa_circ_0009000) had a higher level of expression in SLE patients. We further analyzed 62 patient samples and 62 healthy samples. As shown in Fig. 1a, circGARS showed significant higher expression levels in SLE patient samples than in healthy samples. CircGARS is a 254-nt circRNA, derived from the backsplicing of pre-RNA of the GARS gene, involving exons 11-12, as shown in Fig. 1b. The back-spliced junction point of circGARS was verified by sanger sequencing (Fig. 1c). Stability was measured using exonuclease RNase R, circGARS was highly resistant to RNase R digestion, whereas the linear RNA of GARS and β-actin was highly resolved (Fig. 1d). FISH results showed that circGARS was mainly localized in the cytoplasm (Fig. 1e). To evaluate the potential of circGARS as a biomarker for SLE, data on clinicopathological parameters were collected in 62 patients with SLE, and the correlation between these parameters and circGARS expression level was tested. Patients with high levels of circGARS had a significantly higher SLEDAI (Fig. 1f) and lower complement C3 levels (Fig. 1g). To evaluate the diagnostic value of circGARS for SLE, we performed receiver operating characteristic (ROC) curve analysis in 62 SLE patients and healthy controls to distinguish between SLE patients and HC. The area under the curve (AUC) was 0.8932 (95% CI = 0.8369–0.9495) (Fig. 1h).

### CircGARS functions as a miR-19a sponge

**Fig. 2:**
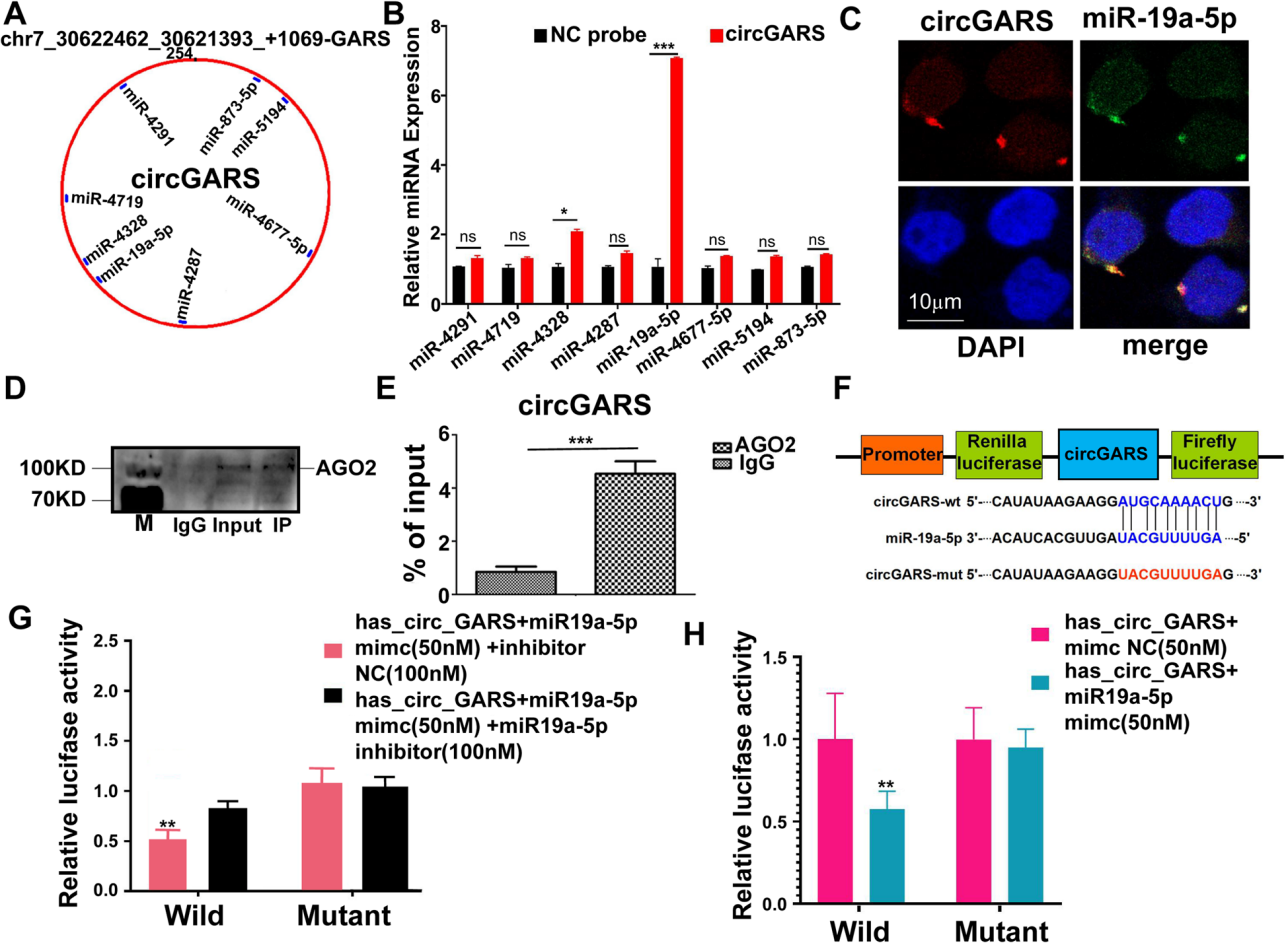
CircGARS functions as a sponge for miR-19a-5p. **a** The ceRNAs were identified using circMir1.0 software. **b** The circGARS probe could capture miR-19a-5p by RNA pull-down analysis. **c** FISH showed the colocalization between circGARS and miR-19a-5p in PBMCs (red: circGARS probes were labeled with Cy3; blue: nuclei were stained with DAPI; green: locked nucleic acid miR-19a-5p probes were labeled with Dig; scale bar: 10 μm). **d** The interaction of circGARS with miR-19a-5p was tested by RIP of AGO2 from HEK293T cells, and the IP efficiency of the AGO2 antibody is displayed by western blotting, which represents circGARS levels associated with AGO2 relative to an input control. IgG antibody served as a control. **e** CircGARS levels were quantified by qRT–PCR, and (IP)/input ratios were plotted by Student’s *t* test. **f** CircGARS contains one site complementary to miR-19a-5p, as analyzed by using the RNAhybrid bioinformatics program. Schematic illustration of circGARS-WT and circGARS-MUT luciferase reporter vectors. **g**, **h** Luciferase reporter assay was applied to verify the interaction between circGARS and miR-19a-5p. The Luciferase activity was normalized to the value obtained in cells transfected with NC oligonucleotides. NC, negative control; miRNA, microRNA; IgG, immunoglobulin G; RIP, RNA immunoprecipitation; qRT-PCR, quantitative real time polymerase chain reaction. All data are presented as the means ± SD of 3 independent experiments (*n* = 3). NS: no significance, **P < 0.01; ***P < 0.001

To discover the potential functional mechanism of circGARS, we used circMir1.0 software (http://www.bioinf.com.cn/) to analyze the potential target miRNAs that could bind with circGARS (Fig. 2a). Subsequently, we purified the circGARS-bound RNA complexes and confirmed the enrichment of miR-19a-5P. RNA pulldown assays suggested that miR-19a-5p may directly bind to circGARS (Fig. 2b). The regulatory effects of miR-19a on TNFAIP3 (A20) and NF-κB signaling have been reported [19, 20]. A20 is a ubiquitin-editing molecule, and it inhibits NF-κB activation and TNF-mediated apoptosis. This protein is involved in cytokine-mediated immune and inflammatory responses. Diseases associated with TNFAIP3 include autoinflammatory syndrome, especially SLE [21]. FISH analysis confirmed that miR-19a-5p was colocalized with circGARS in the cytoplasm (Fig. 2c). We next preformed argonaut 2 (AGO2) immunoprecipitation to determine whether circGARS served as a platform for AGO2 and miR-19a-5p. The results of AGO2 RNA-binding protein immunoprecipitation (RIP) assay (Fig. 2d) and qRT-PCR (Fig. 2e) supported this observation. To further test whether miR-19a-5p is a target of circGARS, wild-type or mutant circGARS was cloned into a luciferase vector (Fig. 2f) and then co-transfected with miR-19a-5p mimics and inhibitor into 293T cells. The repeated dual luciferase experiments showed that miR-19a-5p significantly decreased the luciferase signal of the wild-type circGARS reporter (Fig. 2g, h). The results demonstrated that circGARS binds with miR-19a-5p.

### MiR-19a expression was downregulated and correlated with clinicopathological characteristics in patients with SLE

**Fig. 3:**
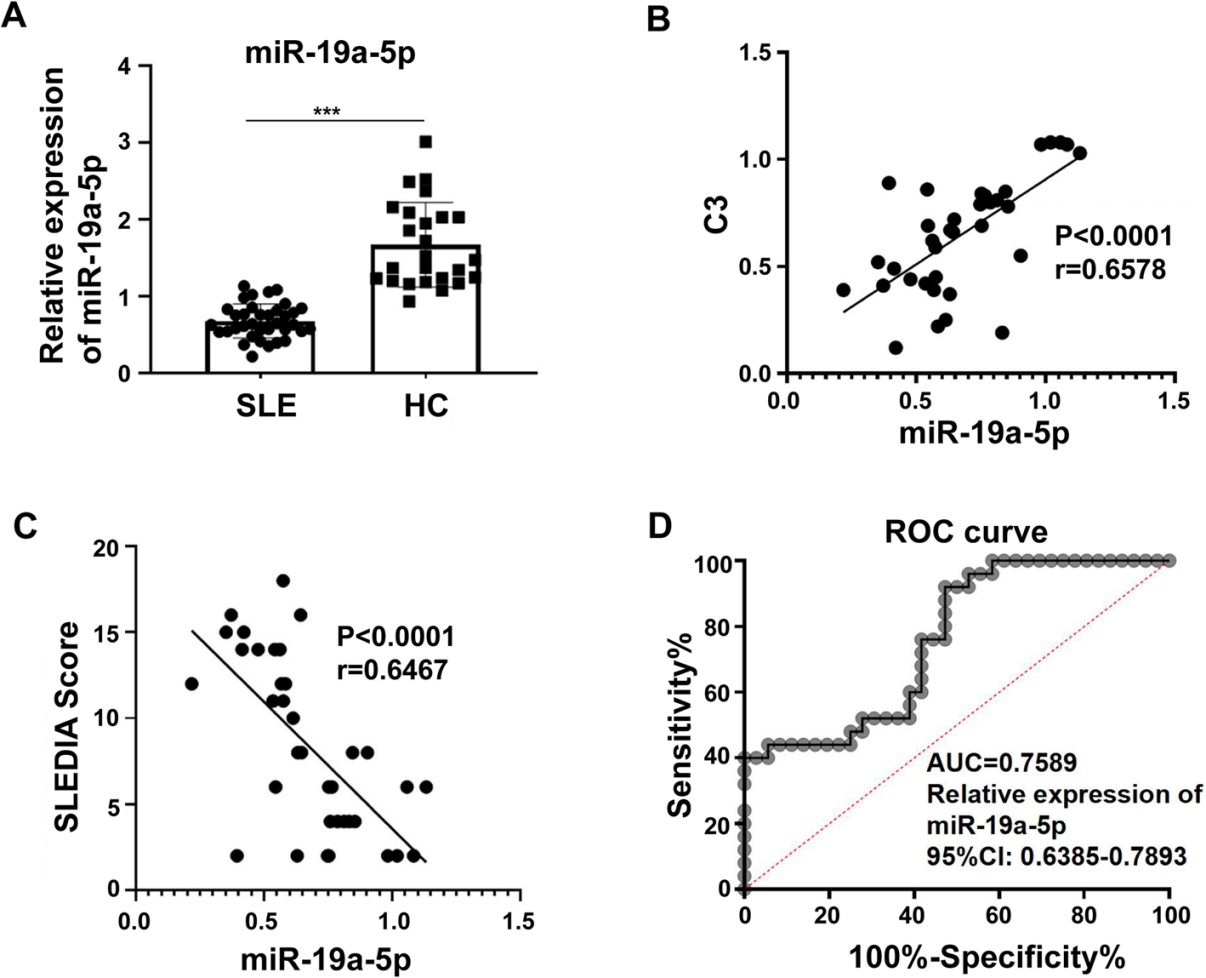
MiR-19a-5p expression was downregulated in SLE patients and correlated with the disease activities of SLE. **a** Expression of miR-19a-5p in PBMCs of 36 patients with SLE and 25 healthy controls. **b**, **c** Correlations between the expression of miR-19a-5p and the SLEDAI and complement C3 levels were analyzed. **d** ROC curve of relative miR-19a-5p expression for distinguishing the 36 patients with SLE from 25 healthy controls. The results are represented as the mean ± SD (*n* = 3). ***P < 0.001

To investigate the role of miR-19a-5p in SLE, we used qRT-PCR to detect the expression of miR-19a-5p in PBMCs of 36 SLE patients and 25 healthy controls. The results demonstrated that the expression of miR-19a-5p was significantly downregulated in SLE (Fig. 3a). We examined the correlation between disease activity and miR-19a-5p expression, and the results showed that there was a strong inverse correlation between miR-19a-5p expression and the SLEDAI in SLE patients (Fig. 3b). miR-19a-5p expression was also positively correlated with C3 levels (Fig. 3c). In order to evaluate the diagnostic value of miR-19a-5p in SLE, ROC curves on relative expression of miR-19a-5p in 36 patients and 25 healthy controls were also analyzed. The AUC was 0.7589 (95% CI = 0.6385–0.8793) (Fig. 3d). These results indicated that the expression of miR-19a-5p related to the disease activity of SLE, and miR-19a-5p could act as a biomarker to evaluate the activity of SLE and validate the effectiveness of SLE treatment.

### CircGARS sponged miR-19a to suppress the expression of the m6A reader YTHDF2 to regulate A20 degradation

**Fig. 4:**
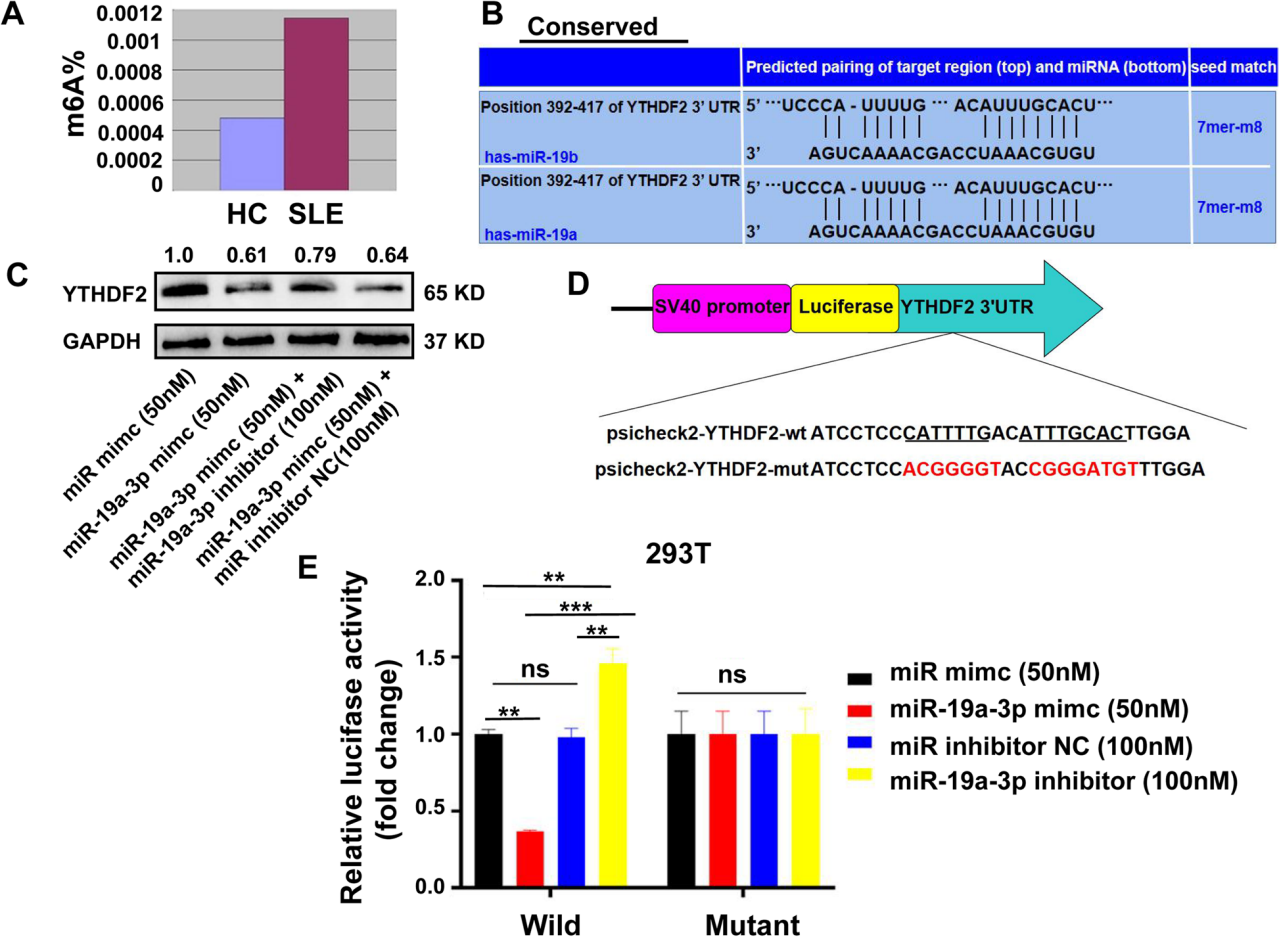
MiR-19a-3p regulated the expression of YTHDF2 by targeting its mRNA 3′-UTR. **a** The degree of global m6A RNA methylation level of total RNA in 36 SLE samples was higher than that of the 25 healthy controls (*P* < 0.05). **b** Predicted the potential miRNAs targeting the 3′-UTR of YTHDF2 by TargetScan, and miR-19a had binding sites for YTHDF2. **c** WB (Western blot) analysis demonstrated that miR-19a-3p could significantly inhibit the expression of YTHDF2 in PBMCs, suggesting that miR-19a-3p is involved in the regulation of YTHDF2 mRNA. **d** Schematic diagram of the binding sites of miR-19a-3p in the 3′-UTR of YTHDF2 mRNA. The mutant was produced at the 3′-UTR of YTHDF2 mRNA. The 3′-UTR sequence of the YTHDF2 mRNA containing the wild-type (or mutants) of the miR-19a-3p binding sequence was cloned into the downstream vector of the psicheck2-control luciferase reporter gene. **e** The interaction of YTHDF2 with miR-19a-3p was tested by luciferase reporter assay. Luciferase reporter gene assays was to measure the effect of miR-19a-3p on reporters, such as psicheck2-YTHDF2-wt and psicheck2-YTHDF2-mut in 293T cells. The experiment was repeated at least three times. The results are represented as the mean ± SD (*n* = 3). ns, no significance, **P < 0.01; ***P < 0.001

Although we already know a lot about the m6A modification, the association between m6A modification and SLE remains to be clarified. To investigate whether m6A modification may be involved in SLE etiology and participated in the disease progress of SLE, we analyzed the degree of global m6A RNA methylation level in SLE patients, and the m6A RNA methylation level was significantly higher than that of the control group (Fig. 4a). The abnormal expression of m6A modification proteins may directly affect the global m6A level and thus lead to the alteration of the expression of key immune-related genes and the occurrence of SLE. Therefore, examining the expression of proteins responsible for m6A modification should provide insight on this possible mechanism. In addition, several miRNAs regulate the pathogenesis of SLE and show potential in the treatment of SLE. YTHDF2, an m6A reader protein, plays an important biological role in the regulation of mRNA m6A [24]. YTHDF2 selectively recognizes mRNA m6A to regulate mRNA stability, and the degradation of mRNAs can lead to the decrease of m6A levels [25]. We performed bioinformatics analysis to predict the potential miRNAs targeting the 3′-UTR of YTHDF2 by using the website TargetScan (www.targetscan.org/). MiR-19a was one of the candidates that was predicted (Fig. 4b) and conserved on the website. The Western blot analysis showed that miR-19a-3p could significantly inhibited the expression of YTHDF2 in PBMCs (Fig. 4c), suggesting that miR-19a-3p is involved in the regulation of YTHDF2 mRNA. Therefore, we concluded that YTHDF2 may be one of the target genes of miR-19a-3p. MiR-19a-3p directly targets the 3′-UTR of YTHDF2 mRNA. In the following, we attempted to detect whether miR-19a-3p could regulate YTHDF2 expression at the posttranscriptional level. We cloned the 3′-UTR of YTHDF2 mRNA (called psicheck2-YTHDF2-wt) and its mutant (called psicheck2-YTHDF2-mutant) as shown in Fig. 4d. Luciferase reporter gene assays revealed that miR-19a-3p could weaken luciferase activity by targeting the 3′-UTR of YTHDF2 mRNA. And no decrease in luciferase activity was observed when the target sites were mutated in 293T cells. However, the miR-19a-3p inhibitor resulted in the opposite results (Fig. 4e). Bioinformatics analysis and dual luciferase reporter assays indicated that YTHDF2 was one of the conservative targets of miR-19a-3p. Western blotting assay further demonstrated that miR-19a also downregulated the expression of YTHDF2. These results suggest miR-19a might suppress the expression of YTHDF2. Normally, YTHDF2 can identify mRNA m6A sites to regulate mRNA degradation and increase the m6A levels of mRNAs. Taken together, we speculated that circGARS may regulate m6A modification through miR-19a in SLE.

### CircGARS directly interacted with m6A-associated proteins to regulate the A20/NF-κB signaling pathway

**Fig. 5:**
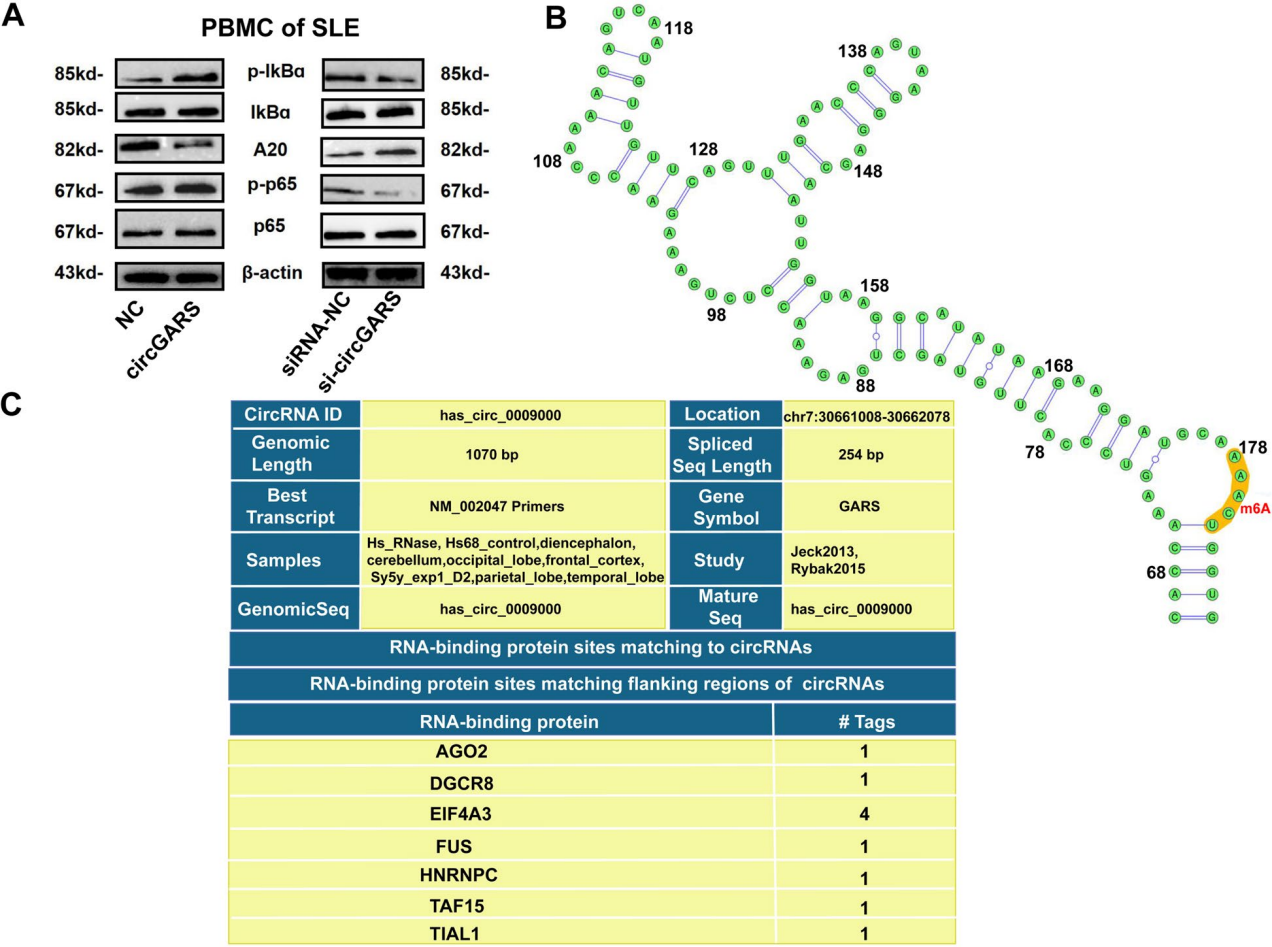
CircGARS directly interacted with m6A-associated proteins to regulate the A20/NF-κB signaling pathway. **a** Western blot analysis of A20/NF-κB signaling-related proteins in PBMCs transfected with circGARS overexpression plasmids, empty vector (NC) si-circGARS, and siRNA-NC in PBMCs of SLE. The results are presented as the mean ± SD (*n* = 3). **b** Image showing the secondary structure of the position of m6A sites located in circGARS. **c** RNA-binding proteins matching to circGARS

To explore whether circGARS could regulate the A20/NF-κB signaling pathway, we transfected circGARS overexpression plasmids into PBMCs of SLE patients. The results revealed that the overexpression of circGARS inhibited the expression of A20 and promoted the phosphorylation of p65 and IkBα. Downregulation of circGARS leaded to decreased phosphorylation of p65 and IkBα and thereby increased expression of A20 (Fig. 5a). These data demonstrated that circGARS regulates the ubiquitin-editing enzyme A20 to activate the NF-κB pathway-mediated immune inflammatory response in SLE.

Cytoplasm-localized circRNAs may be involved in translational regulation through competing endogenous RNAs or RNA-binding proteins (RBPs) interactions. Next, we used the SRMAP (http://www.cuilab.cn/sramp) database and detected an m6A motif in circGARS. The secondary structure schematic diagram demonstrated that the motif was located at position 180 (Fig. 5b). Then, we used the database CircInteractome (https://circinteractome.nia.nih.gov/index.html) and searched RNA-binding proteins matching circGARS (Fig. 5c). The m6A modification protein (HNRNPC) and AGO2 interacted with circGARS, which helps to clarify and understand the role of m6A modification in regulating gene expression of circGARS to a certain extent. Therefore, circGARS directly interacted with m6A-associated proteins to regulate the A20/NF-κB signaling pathway.

## Discussion

With the development of high-throughput sequencing technology, more and more attention has been paid to the function of circRNAs. Many reports have shown that the expression of circRNA is closely related to the occurrence of human diseases, especially tumorigenesis [26, 27]. The recent discovery of thousands of circRNAs and their new function in the regulation of gene expression has led us to investigate their role in SLE.

In this study, we screened an upregulated circRNA called circGARS in SLE on the results of RNA-seq and qRT-PCR analysis. We showed that circGARS expression was upregulated in SLE PBMCs and that the expression of circGARS was also correlated with the disease activity of SLE. Therefore, circGARS may be used as a potential biomarker for SLE.

CircRNA is a new type of non-coding RNA with a covalent closed loop structure. In recent years, many exonic and intronic circRNAs have been discovered in eukaryotes, suggesting that circRNAs are not simply aberrantly spliced by-products but rather have a variety of potential biological functions. The biological functions of circRNAs have been thoroughly studied. CircRNAs can competitively bind miRNAs and regulate the activity of miRNAs on other target genes. For example, circPSMC3 can be used as a new potential biomarker for detecting gastric cancer (GC). CircPSMC3 plays role in the progression of GC via sponging miRNA-296-5p to modulate the expression of PTEN [28]. Exonic circRNA CDR1as/ciRS-7 contains 63 or 70 binding sites for miR-7 and is a powerful miRNA sponges [29]. More and more evidence has also supported the biological function of circRNAs, such as circRNAs regulate gene expression by interacting with RNA-binding proteins (RBPs), interfering with microRNA activity and signaling pathways. CircRNAs can also be translated and function as encoded proteins. In addition, circRNAs have potential applications in regulating immune response and cell proliferation as well as in biomedical research. For example, the overexpression of circRNAs containing dsRNA in PBMCs or T cells in SLE mitigates abnormal PKR activation cascades [30, 31].

Here, bioinformatics analysis showed that circGARS shares the MRE of miR-19a-5p, and luciferase reporter gene assays and RIP experiment verified the direct interaction between circGARS and miR-19a-5p. Further molecular experiments proved that circGARS regulated the ubiquitin-editing enzyme A20 to influence the NF-κB pathway-mediated immune inflammatory response in SLE. Next, we identified that circGARS interacted with the proteins HNRNPC and the m6A motif in circGARS, suggesting that the function of circGARS occurs through m6A-dependent modification. The function of circRNAs has been extensively studied from different angles. Although m6A is considered to be a rich regulator in mRNAs and ncRNAs, and is involved in many aspects of posttranscriptional mRNA metabolism, researches about the effects of m6A modification on cellular circRNA biology are scarce. N6-methyladenosine (m6A), the most abundant internal modification in mRNA, influences the biological regulation of RNA-protein interactions. YTH domain family 2 (YTHDF2) protein can directly recognize m6A and affect the stabilization of cytoplasmic mRNA [32]. Normally, YTH domain family 1 (YTHDF1) can regulate mRNA translation efficiency by recognizing mRNA m6A, whereas YTHDF2 is reported to regulate mRNA degradation and cell viability [33–35]. Yang Z et al. reported that microRNA-145 combined with YTHDF2 modulates m6A levels by targeting the 3′-untranslated mRNA region of m6A, leading to an increase in mRNA methylation [36]. Thus, we further search the mechanism by which circGARS and m6A-dependent modification regulate the expression of genes. Through bioinformatics analysis, we found that YTHDF2 was one of the conservative targets of miR-19a-3p. Herein, dual luciferase reporter assays and western blotting demonstrated that miR-19a shows a distinct regulatory function with YTHDF2 in promoting m6A-modified circGARS, supporting m6A as a potential selection signal for mammalian circRNA metabolism. Our results also demonstrate that YTHDF proteins may function as m6A modification proteins that promote SLE progression by mediating A20 degradation. It is worth mentioning that the recognition of m6A-modified circRNAs by YTHDF2 do not facilitate their degradation but play a part in segregating m6A-circRNA and inhibiting innate immunity [37]. Similarly, the deletion of METTL3 or YTHDF2 has been reported to stabilize IFNB1 in an m6A-dependent manner following viral infection, leading to subsequent suppression of the virus reproduction in host cell [38]. Previous studies have showed that the complexes of YTHDF2-HRSP12-RNase P/MRP and UPF1-G3BP1 may play a key role in regulating the decay of m6A-modified linear mRNAs and degrading mRNAs with a high level of structure in the 3′-untranslated regions [39, 40]. The m6A modification is reportedly widespread in circRNAs [41]. However, their precise function remains unknown.

The function of m6A modification to regulate miRNA processing also makes m6A an attractive research subject in SLE. However, whether other miRNAs can direct AGO2 to degrade circRNAs harboring miRNA-binding site(s) remains unknown. Moreover, several miRNAs are involved in the pathogenesis of lupus and show therapeutic potential in SLE [3, 42–45]. The homeostasis level of some miRNAs is affected by the knockdown of the expression of m6A demethylase FTO [46], suggesting that the signaling pathways concerning m6A modification with miRNAs should be further explored. How does m6A modification regulate miRNA dysregulated in SLE remains to be determined. Whether and to what extent m6A modification leads to miRNA dysregulation in SLE remains to be elucidated. Research has shown that the binding activities of HNRNPC and m6A-switches will modulate the function of RBP, affecting the gene expression and RNA maturation [32]. The biological regulation of m6A interaction with RNA-protein still needs further clarification. Along with the role of m6A modification in gene expression regulation by modulation of translation, mRNA stability, pre-mRNA splicing, RNA structure, and pri-miRNA processing became increasingly clear. Further study on the role of m6A modification and epitranscriptomics in SLE will promote our understanding of the pathogenesis of SLE.

## Conclusions

**Fig. 6:**
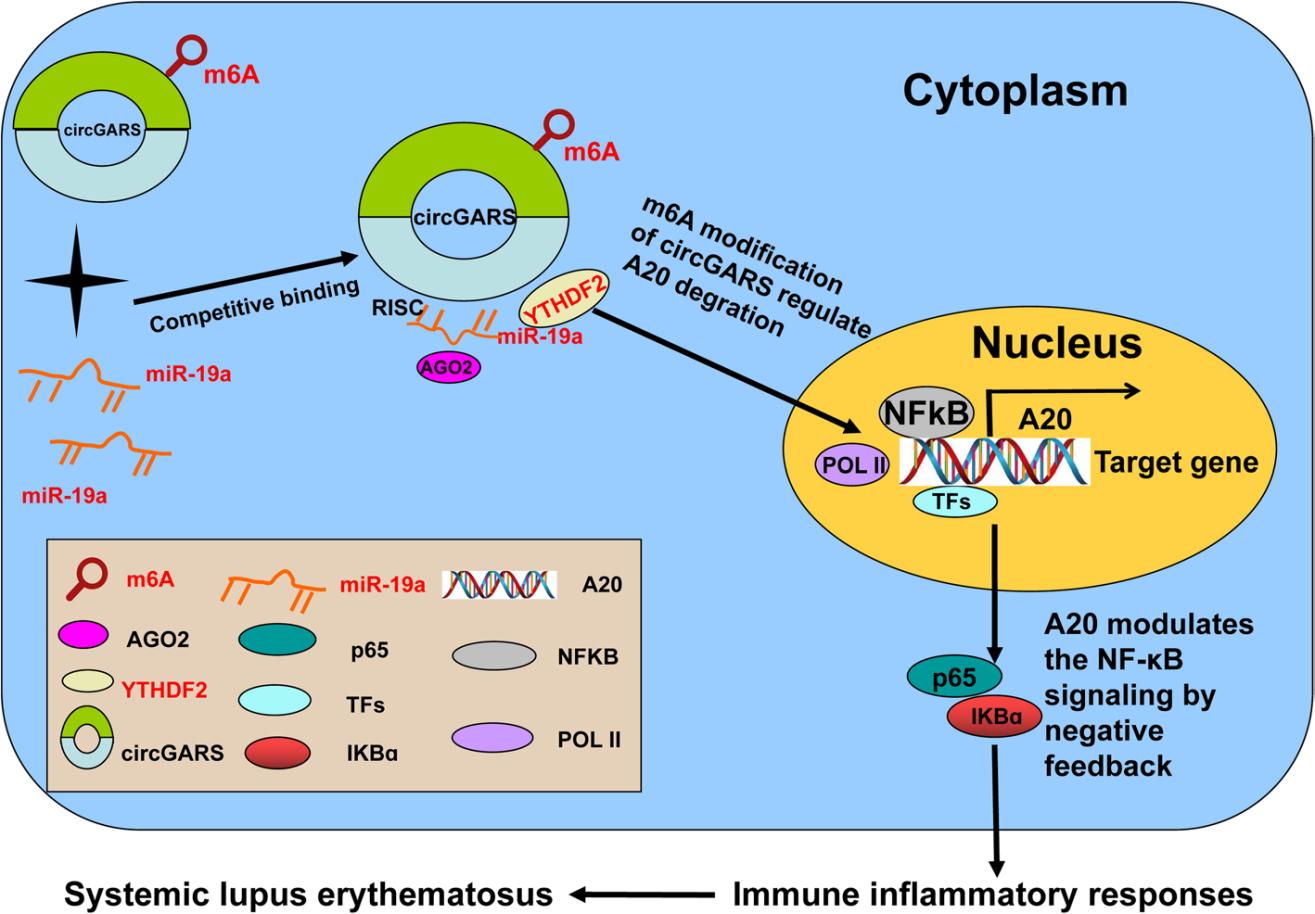
Schematic diagram of the regulatory mechanism of circGARS in the pathogenesis of SLE. CircGARS directly combined with miR-19a regulate the expression of A20 to influence the immune responses regulated by the NF-κB pathway as a negative feedback mechanism. Moreover, circGARS enhanced the transcriptional level by directly binding to m6A modification proteins. In addition, miR-19a regulated A20 degradation by downregulating YTHDF2

Overall, our study discovered a differentially expressed circRNA from patients with SLE, detected the remarkably increased expression of circGARS, and demonstrated that circGARS directly combined with miR-19a-5p. Functionally, circGARS regulated the expression of A20 to influence the inflammatory immune responses regulated by the NF-κB pathway as a negative feedback mechanism. Moreover, circGARS enhanced the transcriptional level by directly binding to m6A modification proteins. In addition, miR-19a-3p regulated A20 degradation by downregulating YTHDF2. We provide the first evidence that circGARS is a key circRNA related to m6A modification in SLE patients and might be a diagnostic/ prognostic biomarker for SLE (Fig. 6).

## Supplementary Information

**Additional file 1: Table S1.** Detailed information on the SLE patients.

**Additional File 2: Table S2.** Detailed information on the SLE patients.

**Additional File 3: Table S3.** Primers for RT-qPCR assays.

**Additional File 4: Table S4.** Primers for RNA pulldown RT-qPCR assays.

**Additional File 5: Figure S1.** Volcano plot showing the selected and identified 10 upregulated circRNAs and 10 downregulated circRNAs significantly related with SLE.

**Additional File 6: Table S5.** Identified and selected 10 upregulated circRNAs and 10 downregulated circRNAs (|fold change|>2, *p*<0.01) between healthy controls and SLE patients by circRNA RNA-seq data.

## Data Availability

The data that support the findings of this study are available from the corresponding author upon reasonable request.

## Abbreviations

SLE: Systemic lupus erythematosus
PBMCs: Peripheral blood mononuclear cells
m6A: N6-methyladenosine
TNFAIP3: Tumor necrosis factor alpha-induced protein 3
MiR-19a: MicroRNA-19a
MiRNAs: MicroRNAs
YTHDF1: YTH N6-methyladenosine RNA-binding protein 1
YTHDF2: YTH N6-methyladenosine RNA-binding protein 2
SLEDAI: Systemic Lupus Erythematosus Disease Activity Index
ROC: Receiver operating characteristic
AUC: Area under curve
LncRNAs: Long noncoding RNAs
NF-κB: Nuclear factor-κB
RBPs: RNA-binding proteins
FISH: Fluorescence in situ hybridization
RIP: RNA-binding protein immunoprecipitation
AGO2: Argonaut 2
