# Supplementary material for: N6-methyladenosine-dependent modification of circGARS acts as a new player that promotes SLE progression through the NF-κB/A20 axis
Source: Arthritis Res Ther. 2022 Feb 4;24:37. doi: 10.1186/s13075-022-02732-x (PMC8815128; doi:10.1186/s13075-022-02732-x)
Supplement: Supplementary file 1 — Additional file 1: Table S1. Detailed information on the SLE patients. [file 13075_2022_2732_MOESM1_ESM.docx]

**Table S1 Clinical features of SLE patients and demographic data of HCs**

| **characteristics** | **SLE patents(n=62)** | **HCs(n=62)** |
| --- | --- | --- |
| **Age, years, median (IQR)** | **37.0(20.8-54)** | **36.0(21.5-53.4)** |
| **Female, n (%)** | **55(89)** | **55(89)** |
| **SLEDAI score, mean (IQR)** | **9.05(2.73-15)** | **N/A** |
| **Complement C3, median (IQR)** | **0.58(0.25-0.92)** | **N/A** |
| **Complement C4, median (IQR)** | **0.14(0.04-0.27)** | **N/A** |

**IQR: interquartile range.**
