# Supplementary material for: N6-methyladenosine-dependent modification of circGARS acts as a new player that promotes SLE progression through the NF-κB/A20 axis
Source: Arthritis Res Ther. 2022 Feb 4;24:37. doi: 10.1186/s13075-022-02732-x (PMC8815128; doi:10.1186/s13075-022-02732-x)
Supplement: Supplementary file 3 — Additional File 3: Table S3. Primers for RT-qPCR assays. [file 13075_2022_2732_MOESM3_ESM.docx]

**Table S3 Primers used in real-time quantitative PCR experiment**

| **RNAs** | **Primers** |
| --- | --- |
| **circGARS** | **F:5'TGCCATTTGTGATGAGTGCT 3'**  **R:5'CAACAATCTCAATCCAACCCCT3'** |
| **GARS** | **F:5'GGCAGCACATGGAGAATGAGATGG 3'**  **R:5'ACAGGAACGATCAGCACATCCAAC3'** |
| **β-Actin** | **F:5'GGTGAGCTGCGAGAATAGCC3'**  **R:5'CTCCGACCAGTGTTTGCCTT3'** |
| **Reverse transcription primer** | **5'GTCGTATCCAGTGCGTGTCGTGGAGTCGGCAATTGCACTGGATACGACXXXXXXXX3'** |
| **U6** | **F:5'TCGCTTCGGCAGCACATA3'**  **R:5'TTTGCGTGTCATCCTTGC3'** |
| **miR-19a-5p** | **F:5'GGGTGTGCAAATCTATGCAA3'**  **R:5'CAGTGCGTGTCGTGGAGT3'** |
