# Supplementary figures and images for: N6-methyladenosine-dependent modification of circGARS acts as a new player that promotes SLE progression through the NF-κB/A20 axis
Source: Arthritis Res Ther. 2022 Feb 4;24:37. doi: 10.1186/s13075-022-02732-x (PMC8815128; doi:10.1186/s13075-022-02732-x)

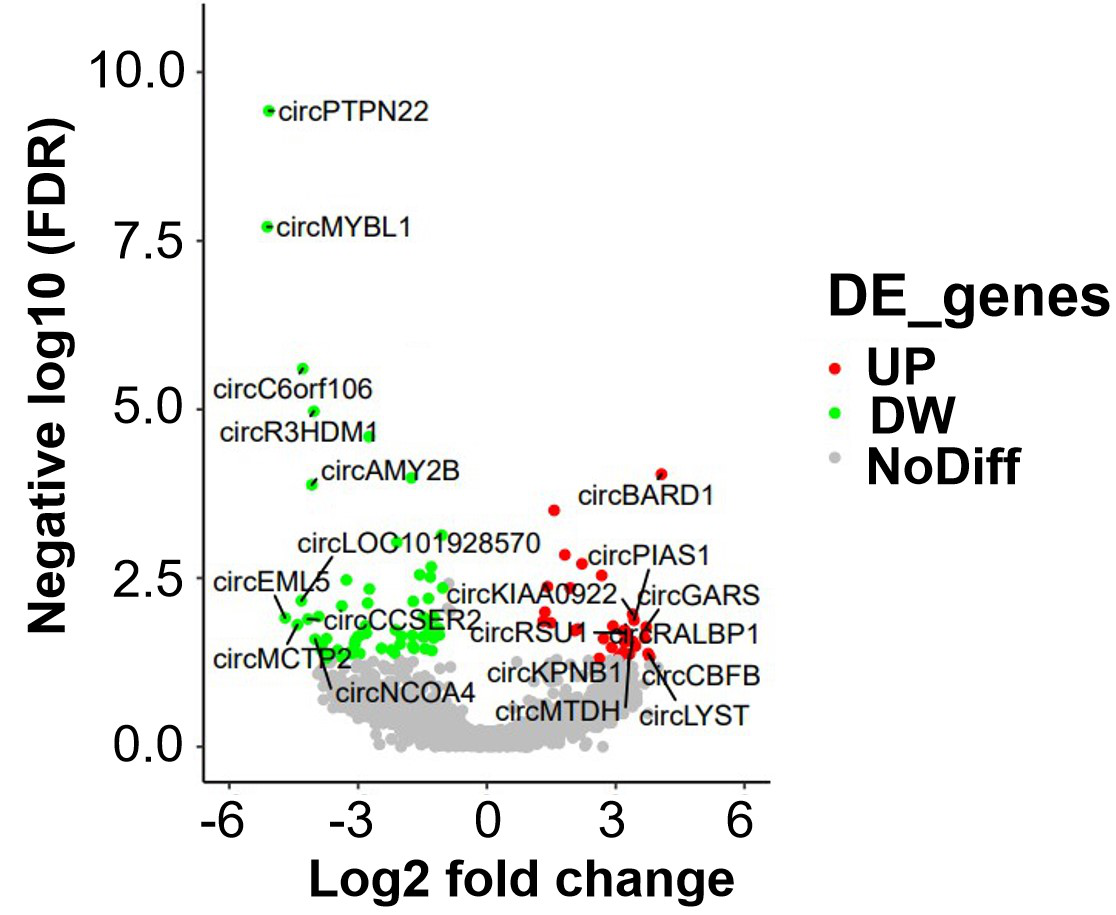

Supplement: Supplementary file 5 — Additional File 5: Figure S1. Volcano plot showing the selected and identified 10 upregulated circRNAs and 10 downregulated circRNAs significantly related with SLE. [file 13075_2022_2732_MOESM5_ESM.tif]
