# Supplementary material for: N6-methyladenosine-dependent modification of circGARS acts as a new player that promotes SLE progression through the NF-κB/A20 axis
Source: Arthritis Res Ther. 2022 Feb 4;24:37. doi: 10.1186/s13075-022-02732-x (PMC8815128; doi:10.1186/s13075-022-02732-x)
Supplement: Supplementary file 6 — Additional File 6: Table S5. Identified and selected 10 upregulated circRNAs and 10 downregulated circRNAs (|fold change|>2, p<0.01) between healthy controls and SLE patients by circRNA RNA-seq data. [file 13075_2022_2732_MOESM6_ESM.docx]

**Table S5 Top ten upregulated and downregulated aberrantly expressed circRNAs in SLE PBMCs.**

| **GeneName** | **Style** | **Length** | **Log2FC** | **FDR** | **start** | **end** | **strand** |
| --- | --- | --- | --- | --- | --- | --- | --- |
| **circMYBL1** | **down** | **1705** | **-5.115477217** | **1.95974E-08** | **66572481** | **66602523** | **-** |
| **circPTPN22** | **down** | **1429** | **-5.078002512** | **3.74294E-10** | **113833110** | **113855049** | **-** |
| **circEML5** | **down** | **470** | **-4.700439718** | **0.012294472** | **88712270** | **88715195** | **-** |
| **circMCTP2** | **down** | **275** | **-4.415037499** | **0.015566341** | **94440175** | **94458246** | **+** |
| **circLOC101928570** | **down** | **435** | **-4.321928095** | **0.006856743** | **77271339** | **77273307** | **-** |
| **circC6orf106** | **down** | **548** | **-4.289506617** | **2.47287E-06** | **34606554** | **34646798** | **-** |
| **circCCSER2** | **down** | **450** | **-4.169925001** | **0.01281405** | **84417770** | **84438707** | **+** |
| **circAMY2B** | **down** | **1307** | **-4.078002512** | **0.000131076** | **103565434** | **103575540** | **+** |
| **circR3HDM1** | **down** | **722** | **-4.029747343** | **1.0622E-05** | **135631717** | **135639122** | **+** |
| **circNCOA4** | **down** | **1218** | **-4** | **0.025516196** | **46010222** | **46013639** | **-** |
| **circBARD1** | **up** | **1261** | **4.06608919** | **9.10507E-05** | **214767481** | **214797117** | **-** |
| **circLYST** | **up** | **664** | **3.794415866** | **0.046897945** | **235677090** | **235697272** | **-** |
| **circCBFB** | **up** | **361** | **3.754887502** | **0.041641745** | **67036638** | **67082339** | **+** |
| **circGARS** | **up** | **254** | **3.709658248** | **0.016824184** | **30621392** | **30622462** | **+** |
| **circRALBP1** | **up** | **294** | **3.672425342** | **0.024350113** | **9524593** | **9525851** | **+** |
| **circPIAS1** | **up** | **359** | **3.426264755** | **0.013329489** | **68141945** | **68146700** | **+** |
| **circKIAA0922** | **up** | **283** | **3.392317423** | **0.010872712** | **153626146** | **153632838** | **+** |
| **circKPNB1** | **up** | **528** | **3.392317423** | **0.02747368** | **47663088** | **47668410** | **+** |
| **circMTDH** | **up** | **499** | **3.321928095** | **0.042272963** | **97686667** | **97691188** | **+** |
| **circADCY9** | **up** | **369** | **3.087462841** | **0.044939241** | **3979115** | **3983440** | **-** |
